# PeerWise and Pathology: Discontinuing a teaching innovation that did not achieve its potential

**DOI:** 10.15694/mep.2020.000027.2

**Published:** 2020-10-14

**Authors:** Christopher Dimick Smith, Anya Dai, Diane Kenwright, Rebecca Grainger

**Affiliations:** 1 University of Otago; 2 University of Otago; 3 The Royal New Zealand College of General Practitioners; 4 The Royal New Zealand College of General Practitioners

**Keywords:** MCQ, PeerWise, Multiple-choice questions, constructivism, deep learning

## Abstract

This article was migrated. The article was marked as recommended.

Introduction

Writing and answering multiple choice questions (MCQs) is a learning activity that potentially engages deep learning. We conducted three year-long case studies of MCQ writing and answering in PeerWise to engage students in learning Pathology.

Methods

Overall, an instrumental case-study design with the structure of sequential multiple case studies was used. Across three years fourth year medical students were required to write and answer MCQs. In 2016 students were provided with advice for writing questions and were encouraged to adhere to Bloom’s taxonomy. In 2017, to reduce cognitive load, students were provided with a MCQ template and allocated topics. In 2018, to encourage engagement, students were informed that the top forty MCQs would be in the final exam.

Results

An evaluation survey was used to measure each student’s perception of the MCQ exercise. In 2016 most students had a negative opinion of the MCQ exercise. Students found writing MCQs too time consuming and demanding. In 2017 student’s attitudes to the MCQ exercise were more positive. In 2018 there were insufficient responses to the survey but informal student feedback suggested the MCQ exercise was considered an inefficient use of student study time.

There were minimal changes in student’s activity levels from 2016 to 2017. However, in 2018 when students were informed that the top forty MCQs generated would be included in their final exam they answered a greater number of MCQs than in previous years.

Conclusions

Providing students with templates and assigning topics for MCQs may improve student attitudes toward MCQ writing and including student generated MCQs in the final exam encourages students to answer more MCQs. However, due to high demands on their time, medical students’ prioritised efficiency and MCQ writing may not be an efficient strategy for deep learning.

## Introduction

Multiple choice questions (MCQs) are a common assessment format in medical education. Students therefore often use MCQs for learning, to test understanding and recall ( Burk-Rafel, Santen and Purkiss, 2017; Wynter *et al.*, 2019). MCQ writing for question banks has also been reported as a small group learning activity for medical students, who report this as an enjoyable and useful learning activity ( Gooi and Sfommerfeld, 2015; Harris *et al.*, 2015; Lawrence, Famokunwa and Ziff, 2016). However MCQ writing may be less efficient than other learning methods ( Kurtz *et al.*, 2019) and have high cognitive load for students ( Grainger *et al.,* 2018). Our pilot of an MCQ authoring and answering exercise had variable student acceptance ( Grainger *et al.*, 2018). We now report three consecutive year-long case studies of a student-generated MCQ learning exercise that aimed to enhance deep learning and knowledge construction in a 4 ^th^ year pathology course and reflect on the lessons learnt.

We initially adopted student MCQ writing as this aligned with constructivist principles that underpin our teaching philosophy; that is an emphasis on the importance of providing the learner with opportunities to actively construct their own learning through discovery, reflection, and peer and teacher interaction ( Krause, Bochner and Duchesne, 2012). Through identifying relevant knowledge, constructing a stem, correct answers and plausible distractors students are active participants in their own learning and need to monitor and reflect on their understanding ( Brandon and All, 2010; Krause, Bochner and Duchesne, 2012; Bonasso *et al.*, 2015). We implemented the writing and answering of MCQs using PeerWise, a free website that enables a class to create, answer and review multiple choice questions ( Denny, Luxton-Reilly and Hamer, 2008).

PeerWise has been widely used in higher education with published evaluations being largely favourable. Students studying engineering, computing and medicine are reported to write high quality MCQs ( Denny, Luxton-Reilly and Simon, 2009; Purchase *et al.*, 2010; Walsh *et al.*, 2018) and engagement with PeerWise has been correlated with exam grades even when controlling for initial student ability ( Sykes, Denny and Nicolson, 2011; Bates, Galloway and McBride, 2012; Kay, Hardy and Galloway, 2019). Furthermore students generally report a positive attitude towards PeerWise for learning ( Denny, Hanks and Simon, 2010; Sykes, Denny and Nicolson, 2011; McKenzie and Roodenburg, 2017) and in some settings students create and answer more questions than the minimum required ( Denny *et al.*, 2008; Hardy *et al.*, 2014).

Our aim was to explore student engagement with an MCQ exercise that encourages deep learning and knowledge construction. Consistent with the principles for good quality case study design and reporting ( Cheek *et al.*, 2018), we established a sequential, multiple-case design and used multiple sources of evidence to evaluate our case. After a pilot in 2016 ( Grainger *et al.*, 2018) we adjusted the MCQ exercise, with the aim to improve the student learning experience. In 2017, we aimed to reduce cognitive load by providing a template and assigning specific topics to each student for MCQ creation and in 2018 we aimed to improve student engagement by including 40 student-authored MCQs in the final exam. We report student participation and evaluation of the MCQ exercise and reflect on our results. We ultimately discontinued the MCQ exercise due to our reflections.

## Methods

### Study design

We used an instrumental case study design ( Stake, 1995) with the structure of sequential multiple case studies ( Cheek *et al.*, 2018). Although case studies may have limited generalisability, by having a clearly defined research question as a focal point for seeking insight, we can provide some general understanding of phenomena occurring when introducing new learning innovations to a course.

### Setting and participants

Participants were fourth-year medical students of the University of Otago, Wellington. These students had completed a health science foundation year, two years of biomedical sciences with introductory clinical practice and were in the first of three years of clinically-based learning. Participation in the MCQ-exercise was a compulsory part in a year-long pathology course, which covers anatomic pathology, chemical pathology, microbiology and haematology, and contributed to students’ summative grade. Written consent was obtained and participation in the research project was voluntary. The research was approved by the Human Ethics Committee of the University of Otago (D16/423).

### MCQ-exercise

A 30-minute instructional scaffolding session was delivered to students at the start of the pathology course. The focus was how to write high-quality MCQs involving higher-order thinking. Bloom’s Taxonomy was used to compare and contrast recall-based versus higher-order MCQs ( Krathwohl, Bloom and Masia, 1956). The gamification features of PeerWise were not discussed as we aimed to promote peer-learning and collaboration not competition. Fifty-minute class sessions were scheduled for the MCQ exercise, generally within one to two weeks of relevant lectures and small group tutorials.

Students were asked to write eight MCQs focusing on aspects of anatomic pathology. Each MCQ needed a stem, one correct answer and three to four distractors. A justification of the correct answer and an explanation of the distractors was required. Peer feedback evaluating MCQs, by rating and commenting on the MCQ or the explanation was strongly encouraged but not compulsory. The MCQ rating scale has six points, with descriptors of 0 very poor, 1 poor, 2 fair, 3 good, 4 very good, 5 excellent. The PeerWise “Answer Score” gives students 10 points for a correct answer, while two points are deducted for an incorrect answer ( Denny, 2013). Completion of the PeerWise activity contributed 20% of students’ final grade for the course while the 80% balance came from a two hour online examination consisting of 100 single-correct answer MCQs, administered at the end of the academic year.

The student-generated MCQ exercise was included in the course for three years, with each year representing a case study. The course teachers changed aspects of the MCQ exercise each year with an aim to increase the acceptability to students. The details of changes are reported with the results.

### Data collection and analysis

In the week following the closing of the MCQ exercise students were asked to complete a survey. The survey was distributed on paper during teaching time for anatomic pathology by a research assistant. This survey consisted of 26 Likert scale questions (7-item response from 1 strongly disagree to 7 strongly agree) and open-ended survey questions (Supplementary file 1). The Likert scale part of the survey contained questions relating to different aspects of students perception of the MCQ exercise such as educational value, satisfaction and ease of use (see Grainger *et al.*, 2018 or supplementary files for more details). The open-ended questions asked students about aspects of their experience with the MCQ exercise and had minor differences between case studies to capture opinions of interest. Summary statistics for quantitative data analysis were calculated using IBM SPSS (version 25). For data that were not normally distributed a Kruskal-Wallis test was used. Multiple pairwise comparisons had Bonferroni correction applied. Open-ended survey responses were analysed following the procedures of qualitative content analysis by two authors (CS, RG) ( Mayring, 2014, pp. 79 - 87; Schreier, 2014).

## Results

We present overall student participation data for all three case studies together then describe each case study (year) with changes to delivery or requirements and results together.

### Student participants

In each case study the class size was just over 100 students, with a mean age of 22 years (see Supplementary File 2, Table 1). In 2016 and 2017 the response rate to the survey was 58% and 61% respectively. In 2018 the response rate was 16% and demographic data provided by students was incomplete.

### Participation in MCQ authoring, answering and commenting

In all three years most students authored only the requirement of eight MCQs with 81/106 (76%) in 2016, 96/102 (94%) in 2017 and 99/110 (90%) in 2018. In 2016 and 2018 one student did not meet the requirement of authoring eight questions. In comparing participation across the years there was a significant effect of year for MCQ authoring, answering and commenting (all *p’s* < 0.005, see Supplementary File 2, Table 3 for more details). Students authored a greater number of questions in 2016 than other years and pairwise comparisons for the number of questions authored showed significant difference between 2016 (M = 8.3, SD = 1.1) and 2017 (M = 8.1, SD = 0.54, *p <* 0.005), 2016 and 2018 (M = 8.2, SD = 0.9, *p* < 0.05), but no significant difference between 2017 and 2018 ( *p >* 0.5). In 2018, students answered a greater number of MCQs than other years and pairwise comparisons for the number of MCQs answered showed that there was a significant difference (between 2018 (M = 276.6, SD = 193.7, *p* < 0.001) and 2017 (M = 157.7, SD = 106.7) and 2018 and 2016 (M = 152.0, SD = 82.1, *p* < 0.001) and there was no significant difference between 2016 and 2017 ( *p >* 0.5). Finally, there also was a significant difference in the number of comments submitted between 2018 (M = 16, SD = 23.9) and 2017 (M = 12.6, SD = 21.3 *p* < 0.001) and 2018 and 2016 (M = 8.0, SD = 12.2 *p* < 0.001), but there was no significant difference between 2017 and 2016 ( *p* > 0.1).

#### Case study 1: 2016

The pilot has been reported in detail elsewhere ( Grainger *et al.*, 2018). In brief students were required to author two MCQs for each of four topic areas (respiratory, cardiovascular, central nervous system and gastroenterology). The 20% of Pathology grade consisted of 10% for authoring of eight MCQs and 10% for obtaining a PeerWise “Answer score” of at least 800 (meaning a correct answer at least 80 questions). Seven 50-minute sessions were allocated for the MCQ exercise between February and May and the MCQ exercise was not available to students after May. Students completed the survey in the last week of May.

**Figure 1.**
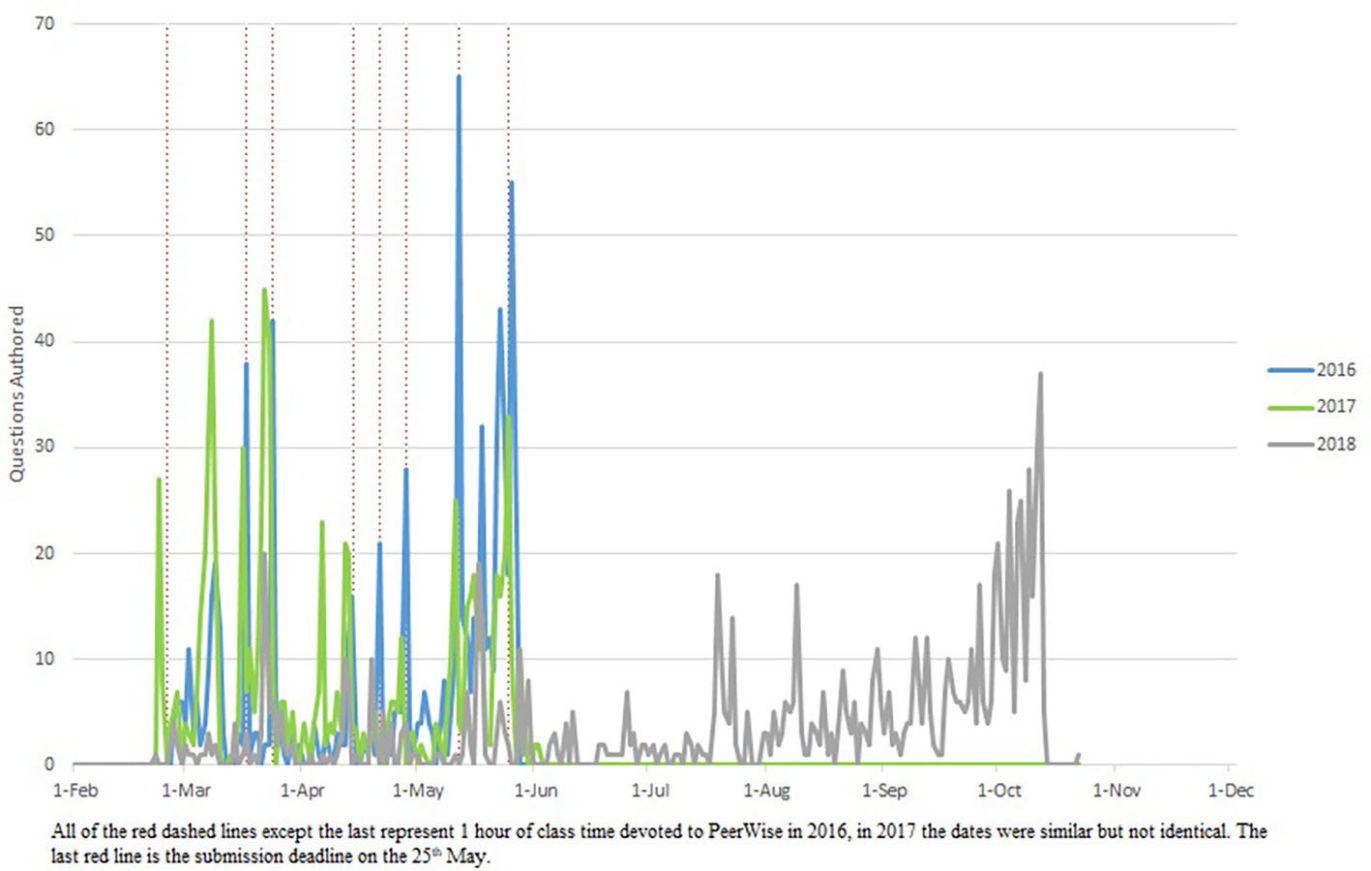
Activity data for authoring PeerWise MCQs.

**Figure 2.**
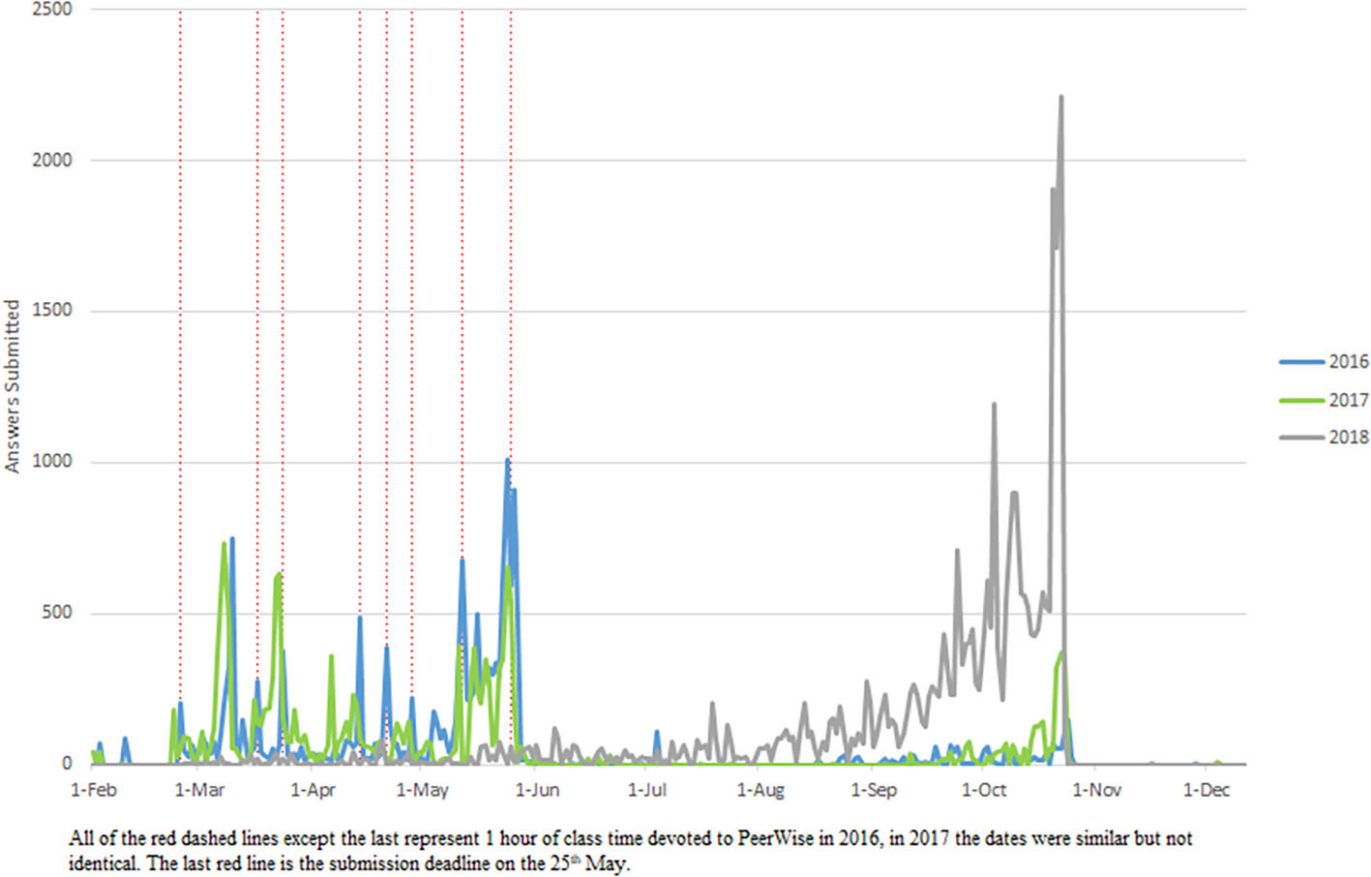
Activity data for answering PeerWise MCQs.

**Figure 3.**
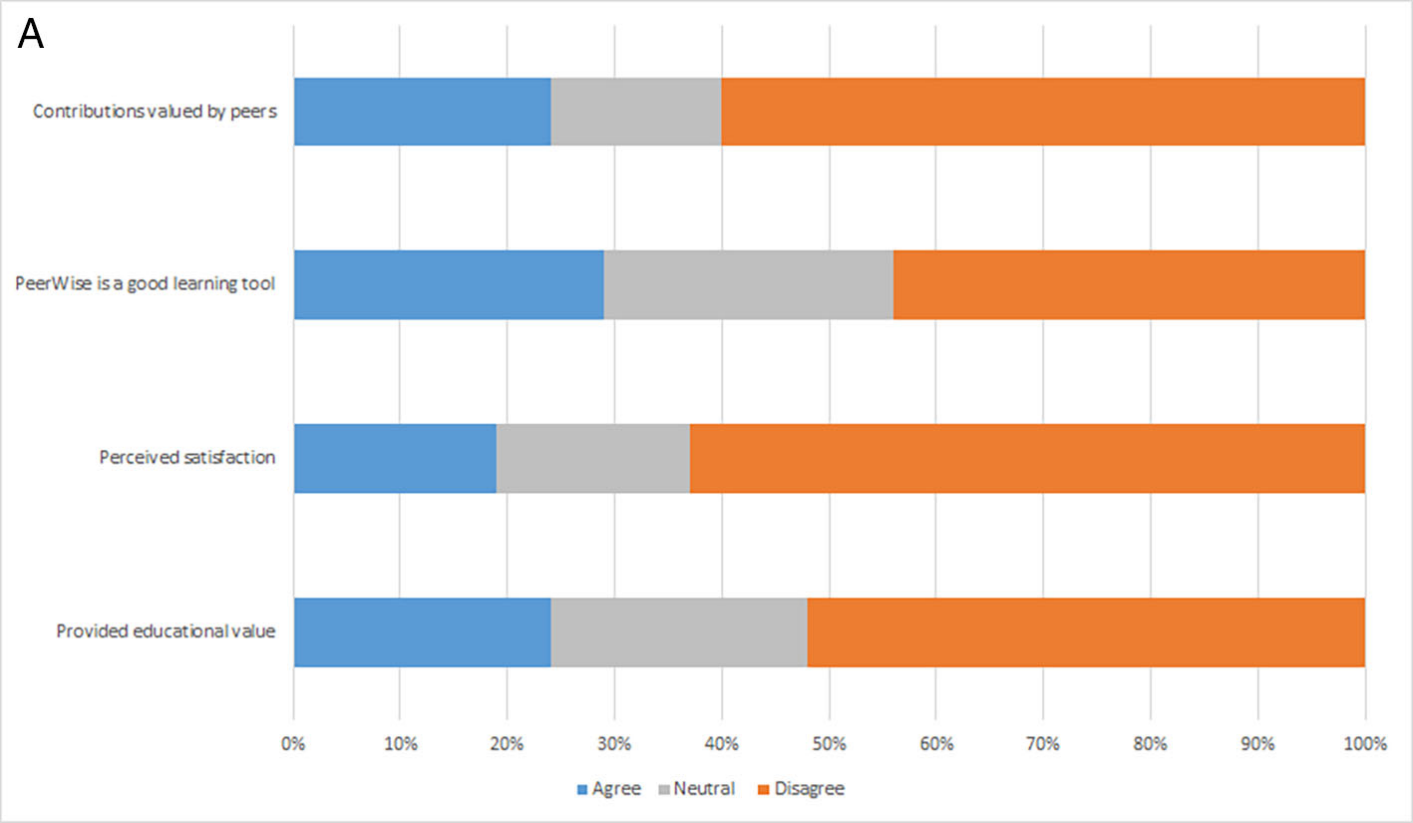

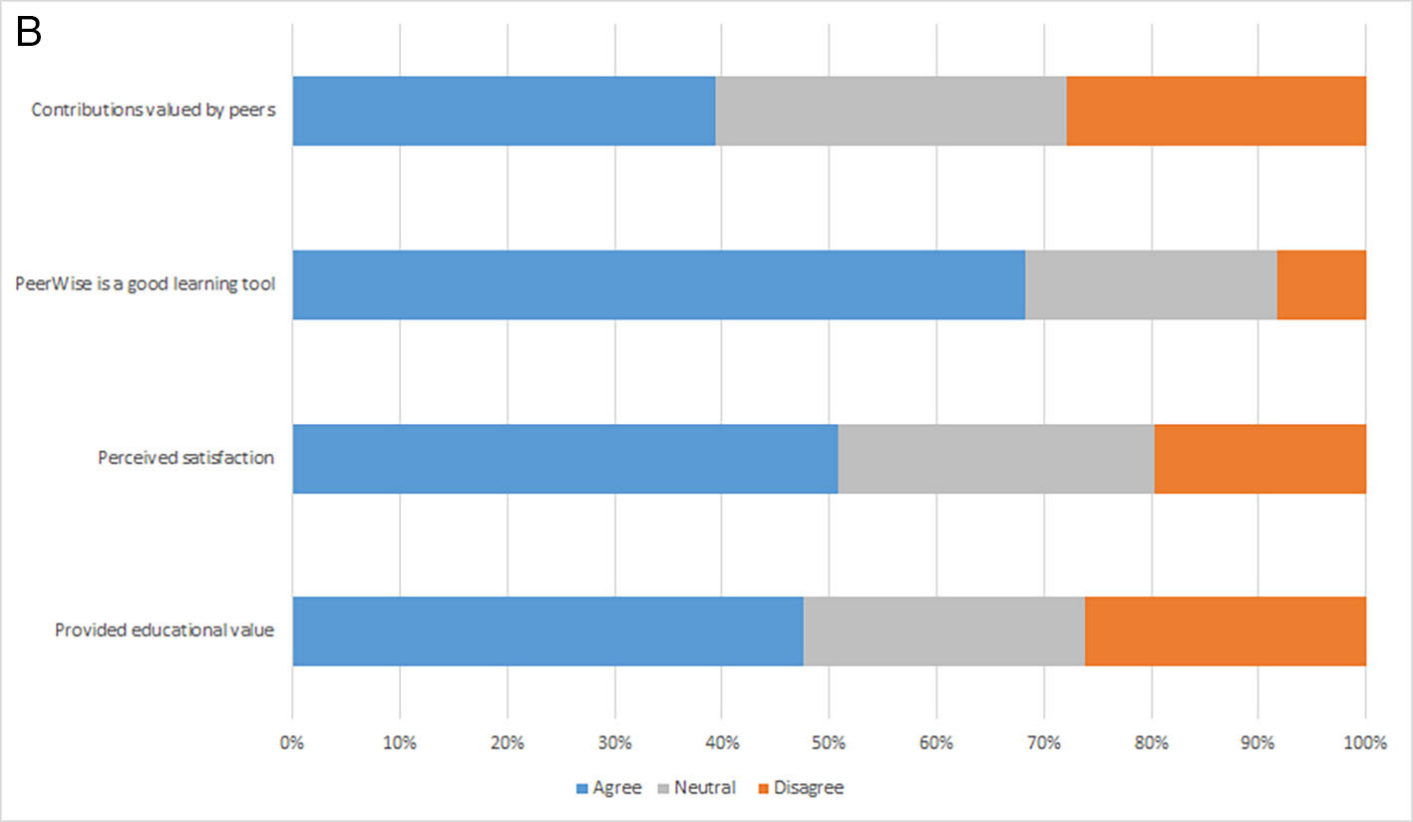
Student Perception of aspects of the MCQ exercise, A 2016, B 2017.

Student MCQ writing ( Figure 1) and answering ( Figure 2) showed bursts of activity, which corresponded to assigned class time. The student survey responses suggest largely negative attitudes towards the MCQ exercise ( Figure 3). Only 24% (15/62) of respondents agreed (responses ranging 5-7) authoring MCQs provided educational value, only 19% (12/62) agreed they were satisfied with the MCQ exercise and only 29% (18/61) agreed that the MCQ exercise is a good learning tool. Additionally, students did not feel their work was valued by their peers with only 24% (15/62) of students agreeing that their peers valued their contributions, nor did many believe that they gained learning benefits by collaborating with peers as part of the MCQ exercise as only 37% (23/62) agreed collaborating with peers was beneficial. The estimated time to author each MCQ was reported as under 30 minutes by 8% (5/62) of students, 30 min to 1 h by 51% (32/62), 1 to 2 h by 26% (16/62) and more than two hours by 15% (9/62).

For the free-text question on the difficulties encountered the most common responses were; MCQ writing was too time consuming (15/42), it was difficult to write a novel question or be creative (8/42) and that they lacked sufficient knowledge to write a question (7/42) (see Supplementary File 2 (Table 4) and Supplementary File 3 for more details). Free-text responses on what should be changed about the exercise were that the MCQ exercise should be optional or discontinued (19/49), no class time should be scheduled for MCQ writing (12/49) and to change the incentives or grading of the exercise (6/49).

#### Case study 2: 2017

In 2017, the goal was to reduce the cognitive load required by the students and improve attitudes towards the MCQ exercise. To reduce the cognitive load imposed by choosing a disease or topic, students were assigned two diseases or named conditions in each of the four topic areas for their MCQ writing, including which aspects of the disease (e.g. pathogenesis, macroscopic appearance, histology). The scaffolding session and supporting written material were adapted; a template aimed to support creation of clinical case-based questions was provided and a worked example was discussed during the scaffolding session. The course assessment requirements did not change, but more class time was allocated to MCQ writing with nine, 50-minutes sessions allocated to the MCQ exercise between February and May. PeerWise was not available to students after May. Students completed the survey in the last week of May.

Again there were bursts of student MCQ writing ( Figure 1) and answering ( Figure 2), which corresponded to assigned class time. Students’ perception of the educational value of the MCQ exercise was more positive. Of the students who completed the survey almost half (29/61, 48%) agreed writing MCQs provided educational value. The majority of students (69%, 42/61) agreed that writing MCQs improved their depth of understanding and 64% (39/61) agreed that MCQs assisted in analysing concepts and knowledge in lectures and tutorials, whereas only 39% (24/61) agreed that the MCQ exercise assisted in understanding how Pathology is applied to clinical practice.

Students were also more positive about other aspects of the MCQ exercise ( Figure 3). Fifty-one percent (31/61) of students agreed they were satisfied with the MCQ exercise, 39% (24/61) agreed that their contributions were valued by their peers and 68% (41/60) agreed that the MCQ exercise was a good learning tool. Again the social learning aspect of the MCQ exercise was not achieved with only 38% (23/61) of respondents agreeing that collaborating with peers was beneficial. Estimated time to author each MCQ as was reported as under 30 minutes by 22% (13/59) of students, 30 min to 1 h by 58% (34/59), 1 to 2 h by 14% (8/59) and more than two hours by 7% (4/59).

In 2017, the most common free-text responses for the difficulties students encountered were difficulty in creating an MCQ following the template on their assigned topic (17/49), the structure and complexity of creating questions (12/49) or that they had no difficulties (10/49). For the free-text question regarding making changes in the activity the most common responses were no changes should be made (7/35), the quality of questions should be improved (6/35) and the incentives or grading should be changed (5/35) (Supplementary File 2, Table 5).

#### Case study 3: 2018

In 2018, the goal was to enhance student engagement with the MCQ exercise and its perceived value. The 40 most highly rated student-generated MCQs would be included in their final exam after editing by faculty. Students were informed of this in the scaffolding session and course materials. As in 2017 students were assigned topics for MCQ authoring and provided with a template. The mark allocation was changed so students could obtain 8% for creating 8 questions (2 for each topic area), 8% of their final grade for answering questions, and 4% for writing eight meaningful comments on other students’ questions. Fifty-minute sessions were assigned to the MCQ exercise on four days between March and May and the MCQ exercise was available to students until the end of October, just before final exams at the end of the academic year.

Students authored ( Figure 1) and answered ( Figure 2) MCQs throughout the year, with a burst of activity in October, most marked for answering. Students answered more questions (M = 276) in 2018 than other years (see the participation subsection and Supplementary File 2, Table 2). Additionally, student’s activity was more distributed with a greater number of days active in 2018 than in 2017 and 2016 (Supplementary File 2, Tables 2 and 3).

The survey was distributed in a class session in October with 18/110 students returning the survey. Many of those returned were also incomplete therefore survey results are not reported. During routine, in-person course evaluation and feedback with faculty students indicated that while they thought that the MCQ exercise was beneficial for learning, many thought that the time required for these benefits was excessive and that other learning methods were more efficient. Faculty (DK) also noted that the student-authored MCQs used in the exam required significant editing to be exam-ready. During faculty course review, it was decided not to include the MCQ exercise in future courses due to patterns of student feedback, student time burden and staff time required to edit student-generated MCQs for exams.

## Discussion

In this sequential, multiple case design study we evaluated the implementation of a student-MCQ writing and answering exercise using PeerWise in a pathology course. We aimed to stimulate active learning and knowledge construction, underpinned by constructivist learning theory. In all three years medical students completed the MCQ exercise. In the third iteration students answered more questions when the PeerWise platform was open to the end of the academic year and the top rated MCQs were edited by faculty and included in the final examination. Student evaluation in the first year was not favourable with most students indicating that they disliked the MCQ-writing exercise in PeerWise and preferred other methods of studying. In the second year templates for MCQ writing and allocation of topics were introduced, with more students reporting positive attitudes about learning achieved through MCQ writing but students reported spending a similar amount of time writing MCQs. In case study three, despite the highest student participation in MCQ answering, there was minimal student participation in the research evaluation of the MCQ-exercise. This is likely to be due to the timing of survey distribution at the end of the year, which was remote from most of the MCQ exercise. The informal student feedback indicating time-burden and lack of efficiency led to a decision not to continue the student-generated MCQ exercise.

There are many explanations for why we observed less positive student responses to MCQ writing and answering in PeerWise than previously reported ( Denny, Hamer and Luxton-Reilly, 2009; Bottomley and Denny, 2011; Sykes, Denny and Nicolson, 2011; McKenzie and Roodenburg, 2017). Our medical students reported a high time burden for each MCQ authored. In a content heavy course, medical students’ may prize efficiency to a greater degree than other students. Furthermore the time taken could suggest that MCQ-writing had a high task complexity for the students and they needed greater instructional support ( Leppink and Duvivier, 2016). In a learning activity where medical students wrote MCQs for assessment in small groups, Kurtz *et al.* (2019) reported the task required a high level of content knowledge, and more problem-solving and content-integration than other study methods. Compared to other implementations of MCQ activities, our course requirements may have been too high for the effort required by students. Students were required to author eight MCQs and answer around 80, whereas other courses have required students to author 2 or 4 questions and answer 10 - 40 questions ( Denny *et al.*, 2008; Denny, Hamer and Luxton-Reilly, 2009; Sykes, Denny and Nicolson, 2011). Our MCQ authoring requirements were determined by the aim to have each student have some engagement with all the anatomic pathology course content. Similarly, in the responses some students indicated that they did not like being required to spend time in class on the MCQ exercise and would have preferred to only use it in their own time. It is possible with less requirements students might have felt more autonomy and shown more positive responses to the MCQ exercise.

Although authoring MCQ questions theoretically contributes to knowledge construction, this may not be an effective *and* efficient learning method for medical students. In an experiment on study strategies using undergraduate students, generating questions led to better memory performance than rereading a text and generating questions led to equivalent performance to answering questions from memory ( Weinstein, McDermott and Roediger III, 2010). However, the time required for generating questions was three or four times longer than required for answering questions or rereading the text, suggesting question writing may not be efficient. Furthermore a recent experimental study suggests that MCQ-writing may be less effective in a limited time period than other learning methods ( Hoogerheide *et al.*, 2019). Undergraduate students were asked to write MCQs or to study and restudy texts, writing MCQs resulted in poorer recall than the re-study condition when students had an 18 minute time limit ( Hoogerheide *et al.*, 2019). It may be the case that other strategies like organising notes and self-testing can promote deep learning while being more time efficient.

We had hoped that students might value the MCQ-writing exercise due to the development of an MCQ bank for examination revision. The high participation in MCQ-answering in the third case study suggests this was at least partially achieved. However, faculty found that even the 40-top rated student-authored MCQs required considerable editing to be exam ready. Previous student developed MCQ banks have involved considerable faculty input for quality improvement ( Gooi and Sommerfeld, 2015; Harris *et al.*, 2015). We would advise implementation of student-MCQ writing for a question bank to have allowances for higher faculty input than we allowed for and have MCQ faculty review built into the programme.

There are a number of limitations in this multiple case-study design. We had a minimum survey non-response of 40% with over 80% of students not responding in the third year. In the final year the survey was distributed in class time at the *end* of the academic year, when students may have been overburdened by course feedback requests and the MCQ-writing task may have been too remote in their minds. Non-responders in student surveys often have more negative or lower ratings than responding students ( Thorpe, 2002; Rudland, Pippard and Rennie, 2005; Kherfi, 2011; Treischl and Wolbring, 2017). It is therefore possible that across the three years students attitudes overall towards PeerWise were worse than suggested by the survey data. Our data is also limited because we did not have permission to use results from the end of year MCQ examination for research or collect some other performance data. It is therefore possible that the MCQ writing and answering exercise was more valuable than students traditional study methods and those who were most active scored higher grades. Past research has identified that PeerWise is associated with better course performance ( Sykes, Denny and Nicolson, 2011; Bates, Galloway and McBride, 2012; Kay, Hardy and Galloway, 2019) and many studies show that students are often inaccurate in judging the effectiveness of learning strategies (e.g., Karpicke, Butler and Roediger III, 2009; Karpicke and Blunt, 2011; Bjork, Dunlosky and Kornell, 2013). Since this was a real-world intervention, we decided that controlling for other factors that might influence examination marks (like student ability etc.) was beyond scope of these case-studies. We undertook a multiple, sequential case study design, which limits the generalisability of the data and means the data is less reliable than if our interventions were experimental or quasi-experimental. Our data, however, does illuminate generalisable principles for other educators, such as the need for careful consideration of task complexity and the instructional support required ( Leppink and Duvivier, 2016) which we underestimated.

Overall, our case studies on MCQ-writing and answering in Peerwise has several takeaways. First, providing additional scaffolding to the MCQ-writing exercise by assigning a topic and providing students with a template seems to make the task easier and improve their attitudes towards it. This may be due to reduced cognitive load. Second, including student-authored MCQs in a final examination appears to increase MCQ answering (practice). This supports the idea of using a question bank to motivate student MCQ engagement with learning. The burden on faculty of editing MCQs to be fit for use in examinations is something that faculty need to consider. Third, even when a learning modality may be effective, student acceptance may require that the task is also efficient. This might be particularly relevant for medical students with high course load and time demands due to experiential learning in the clinical environment. Finally, we report these case studies to illustrate not all teaching innovations achieve the benefits hoped for and that abandoning an underperforming innovation is an option educators must consider.

## Take Home Messages

• MCQ writing might not be a time-efficient learning strategy for medical education.
• Providing templates and assigning topics may reduce cognitive load.
• Including student-generated multiple choice questions in a final exam increases the number of MCQ questions students answer, however, staff may need to edit them before entering them into the final exam.

## Notes On Contributors

Christopher Dimick Smith, PhD, is a research assistant for the Department of Pathology at the University of Otago Wellington.

Anya Dai, PhD, is an educational designer at the Royal New Zealand College of General Practitioners.

Diane Kenwright, MBChB, FRCPA, FNZSP, is an Associate Professor and Head of Department of the Department of Pathology and Molecular Medicine at the University of Otago, Wellington.

Rebecca Grainger, PhD, MBChB, FRACP, is an Associate Professor in the Department of Pathology and Molecular Medicine and Associate Dean of Medical Education at the University of Otago, Wellington
