# Supplementary material for: PeerWise and Pathology: Discontinuing a teaching innovation that did not achieve its potential
Source: MedEdPublish (2016). 2020 Oct 14;9:27. Originally published 2020 Feb 12. [Version 2] doi: 10.15694/mep.2020.000027.2 (PMC10697471; doi:10.15694/mep.2020.000027.2)
Supplement: Peer review history

# Reviewer report: Reviewer response for version 2

**DOI:** 10.21956/mep.20262.r31445

**Published:** 2021-02-17

**Authors:** Richard Hays

**Affiliations:** 1 James Cook University

This review has been migrated. The reviewer awarded 4 stars out of 5 I agree that the reviewers' comments have been addressed. This is a sound paper that is worth reading by all in medical education. Further work is needed: in my experience students often focus on relatively unusual facts rather than application of knowledge so (as with all new MCQs) Item QA is very important. It will be interesting to see further item performance data.

Reviewer Expertise:

NA

No decision status is available

# Reviewer report: Reviewer response for version 2

**DOI:** 10.21956/mep.20262.r31444

**Published:** 2020-10-15

**Authors:** Ken Masters

**Affiliations:** 1 Sultan Qaboos University

This review has been migrated. The reviewer awarded 4 stars out of 5 The authors have addressed the most pressing issues raised by the reviewers, and have nicely rounded off an already solid piece of work.I look forward to seeing further research by the authors in which they have the opportunity to apply the lessons learnt from this innovation, either repeating it differently, or attempting to use the constructivist principles in a different manner.

Reviewer Expertise:

NA

No decision status is available

# Reviewer report: Reviewer response for version 1

**DOI:** 10.21956/mep.19131.r27628

**Published:** 2020-05-17

**Authors:** Ken Masters

**Affiliations:** 1 Sultan Qaboos University

This review has been migrated. The reviewer awarded 4 stars out of 5 An interesting case study on the use (and failure) of PeerWise to generate student MCQs. The authors have clearly laid out their reasonable expectations and the other success stories that led to these expectations. For their description, they appear to have followed a well-considered and well-structured approach. The grade allocation at 20%/80% is a good balance between rewarding good work, and ensuring that the students also have knowledge in other areas.I am very impressed with the amount of data gathered and presented in this case study. It is obvious that the authors have taken great care at gathering as much as possible, and then reacting to it. The change from 2016/7 shows that they took their students’ comments into account as much as reasonably possible, and the 2017 students’ perception reflects that.Overall, the failure does reflect the many complexities and difficulties in any kind of constructivist approach, especially when students are used to an environment where simple learning material for the test has obvious benefits, and the value of constructivist exercises are far more subtle. Most of the value is grounded in philosophical pedagogy, and, if the students are not aware of that, then problems occur. (And, this awareness is not solved by simply telling them, but is a process of internalisation that the students need to experience). When one adds to the this the extra workload placed on the staff, one can see that the process does have many obstacles.Unfortunately, among the many successes of constructivist learning, including PBL and TBL, there are the wrecks, and, and we learn best from those examples that have not gone as we had hoped. While I empathise with the authors, as they have obviously put in a great deal of work to a very good idea, they should also take heart that their paper will help other educators. And that, after all, is the prime goal of medical education publication.The previous reviewer has raised some interesting questions. It might be useful to readers if those could be addressed, either directly, or in a Version 2 of the paper.

Reviewer Expertise:

NA

No decision status is available

# Reviewer report: Reviewer response for version 1

**DOI:** 10.21956/mep.19131.r27629

**Published:** 2020-04-23

**Authors:** Barbara Jennings

**Affiliations:** 1 Norwich Medical School

This review has been migrated. The reviewer awarded 3 stars out of 5 Thank you to the authors for publishing this interesting article which introduced me to the design and use of MCQs via the PeerWise platform. I also appreciated the thorough citation of relevant literature about knowledge construction, question writing, and question banks.The methods used, to evaluate the use of MCQ design to promote active learning of pathology topics, are clearly outlined; and the statistical methodology included correction for multiple comparisons. Some practical details about survey distribution and the timings were not described as fully.The curriculum intervention was evaluated over three academic years – presented longitudinally in this article- and the findings for the first year have been published in a prior case study.The main challenge for me (and I assume for the authors) was drawing generalizable conclusions from three years of data (from a total of 141 students who participated in the evaluation survey) because the parameters for the task had evolved significantly between 2016 & 2018. One particular problem was low uptake of the survey in 2018 because of the particular timings of the survey (noted in the discussion).Minor points: there is no key included in figure 3. A to explain the chart (though it can be inferred from panel B)I have a couple of queries. 40 student-authored MCQs were included in the final exam in 2018 after faculty editing; how many questions were set for that exam in total? Also, gamification was not an aim for the authors but the students seem to have raised it as a suggestion in their open comments – what do the authors think about those student comments? Did the authors and researchers have any aims regarding faculty development (in assessment design) when they designed this pilot curriculum?I respect and appreciate the fact that the authors have taken time to document and discuss a strategy that they ultimately discontinued because of their findings (including the practical & academic challenges of question writing). It is important that negative findings are discussed as fully as positive findings in medical education (as with other fields), avoiding publication bias and duplication of effort.

Reviewer Expertise:

NA

No decision status is available
